# Retraction: Meta-analysis of the synergistic effect of traditional Chinese medicine compounds combined with conventional Western medicine in the treatment of osteoporosis

**DOI:** 10.3389/fendo.2026.1792372

**Published:** 2026-01-28

**Authors:** Frontiers Editorial Office

The Journal retracts the 2025 article cited above.

Following publication, the publisher has found evidence of peer review manipulation. As the scientific integrity of the article cannot be guaranteed and adhering to the recommendations of the Committee on Publication Ethics (COPE), the publisher therefore retracts the article.

This retraction was approved by the Chief Editors of Frontiers in Endocrinology and the Chief Executive Editor of Frontiers. The authors received a communication regarding the retraction and had a chance to respond. This communication has been recorded by the publisher.

